# Brown Lemurs (*Eulemur fulvus*) Can Master the Qualitative Version of the Reverse-Reward Contingency

**DOI:** 10.1371/journal.pone.0048378

**Published:** 2012-10-31

**Authors:** Yannick Glady, Émilie Genty, Jean-Jacques Roeder

**Affiliations:** 1 IPHC DEPE, UMR 7178, CNRS, Strasbourg, France; 2 UDS, Centre de Primatologie, Strasbourg, France; Brain and Spine Institute (ICM), France

## Abstract

Behavioral flexibility that requires behavioral inhibition has important fitness consequences. One task commonly used to assess behavioral inhibition is the reverse-reward task in which the subject is rewarded by the non selected items. Lemurs were tested for their ability to solve the qualitative version of the reverse-reward task with the choice between identical quantities of different food items instead of different quantities of the same food. Two of four subjects mastered the task without a correction procedure and were able to generalize the acquired rule to novel combinations of food. One of the two subjects competent on the quality version of the task could transfer this ability to different quantities of the same food. Our results are compared to lemurs’ performances when tested under the quantitative version in a previous study and those of capuchin monkeys tested under a similar paradigm. The whole results suggest that the qualitative version of the reverse-reward task may be easier to master than its quantitative counterpart and that lemurs perform better than capuchin monkeys as they were able to later transfer the learning rule to the quantitative version of the task.

## Introduction

Self-control has been defined as the ability to delay or suppress a prepotent response in order to maximize benefits. This ability has been studied in a wide range of species including: cleaner fishes (*Labroides dimidiatus* [1]), pigeons (*Columba livia* [2], [3]), rats (*Rattus norvegicus* [4]), sea lions (*Zalophus californianus* [5]) and primates (see below) including humans (*Homo sapiens*): adults [6] and children [7]–[9].

The classical task used to assess self-control abilities is Boysen and Berntson’s reversed reward contingency task [10] which consists of giving the subject a choice between two distinct quantities of the same food. The selection of the smaller quantity results in obtaining the larger and *vice versa*. Many primate species have been tested under this paradigm: prosimians (brown (*Eulemur fulvus)* and black *(E. macaco)* lemurs, [11], [12], [13], New World monkeys (squirrel monkeys (*Saimiri sciureus*) [14], [15], cotton-top tamarins (*Saguinus oedipus)* [16], [17], Old World monkeys (Japanese macaques (*Macaca fuscata*) [18], rhesus macaques (*Macaca mulatta)* [19], white-crowned mangabeys *(Cercocebus torquatus lunulatus)* [20], apes (Gorillas (*Gorilla gorilla)* [21], [22], orangutans (*Pongo pygmaeus)* [21]–[23], bonobos (*Pan paniscus)* [21], [22], chimpanzees (*Pan troglodytes*) [10], [21], [22], [24]–[26]. Most non human primates failed to suppress their prepotent response in the conventional task, often demonstrating chance level performance or worse. However, over-training (increased number of trials) may lead to mastery of the reverse-reward contingency as observed in mangabeys (*Cercocebus torquatus lunulatus)* [20], rhesus macaques (*Macaca mulatta*) [19] and lemurs [13].

All the species listed above were tested in a quantitative version of the reverse-reward task. A study on capuchin monkeys (*Cebus apella*) [27] found better performance when varying kinds of food were first used instead of different quantities of the same food. One group was tested in the conventional quantitative version of the task for 200 trials before being presented with the qualitative version for another 200 trials. A second group was given the qualitative task first and then the quantitative one. Over the first 200 trials, both groups showed an increase in performance, but the one presented with the qualitative task averaged a better score (all monkeys reached at least 75% correct responses by the end of the first phase), whereas the other group remained at chance level (although one of the monkey mastered the quantitative task within these 200 trials). When the conditions were reversed, the “qualitative task first” group did not generalize to the quantitative task, scoring approximately 50% of correct responses. The “quantitative task first” group showed steady improvement on the first 40 trials, but their performance then decreased dramatically and fell below chance level. The single subject that mastered the quantitative task also failed to show positive transfer to the qualitative task.

Lemurs are archaic species close to the common primate ancestor. Therefore, studies on lemurs are worth to clarify our understanding of the emergence of cognitive abilities. Prosimians diverged from the common primate ancestor about 54 million years ago [28] and potentially offer an insight in the ancestral primate mind [29]. Comparing their cognitive abilities with those of monkeys or apes should allow us to clarify the evolutionary constraints (environmental and/or phylogenetic) that have been at work in the past. In order to allow interspecific comparisons, we aimed to test lemurs’ ability to master a qualitative version of the reverse-reward task. We wanted to know if, like capuchin monkeys, lemurs would master this version of the task. Furthermore, we attempted to assess if lemurs, conversely to capuchin monkeys, would generalize the acquired rule when tested with the quantitative version.

## Experiment 1

### Ethics Statement

This study involved the observation of animals without animal handling or invasive experiments done on subjects. This research adheres to the legal requirements of France and to all institutional guidelines. Our study was carried out in full accordance with the ethical guidelines of our institution with the approval of the latter (certificate number: 67–151, French Republic, Bas-Rhin County Hall, French veterinary services).

### Method

#### Subjects and housing

The subjects were four completely naïve brown lemurs (*Eulemur fulvus*) [one adult female, Champagne (4 ys), two adult males Crumble (3 ys) and Clément (7 ys), and one juvenile male, Hiro (1 y)]. All were born and raised at the Centre de Primatologie de l’Université de Strasbourg. Their cages comprised an inside (20°C, 7.6×2.0 m and 2.6 m high for Clément and Hiro, 3.6×2.6 m and 2.6 m high for Crumble and Champagne) and an outside (11.0×3.5 m and 2.6 m high for Clément and Hiro, 3.1×1.7 m and 2.6 m high for Crumble and Champagne) compartment linked by a tunnel allowing isolation of subjects for testing. Both compartments were furnished with tree trunks and ropes. The lemurs had complete freedom of movement between the indoor and outdoor compartments. The lemurs were fed daily with commercial primate pellets and with fruits and vegetables once a week. Food was accessible at anytime during test sessions.

#### Apparatus

The experimental apparatus consisted in a pivoting plastic tray (70×16×25 cm) divided into two equal parts by a central black line. Two white circles (radius: 3 cm) indicated the location of the reward and were centered on each partition of the tray.

#### Procedure

Preferences between the different rewards were assessed before the reverse-reward contingency task was used. Each possible pair of foods from fig, raisin, apple, carrot and commercial primate pellet (the stable lemur diet in the centre) was presented to the subjects, one combination each day in a random order. The different foods were cut into pieces of equal size (approximately 1×1 cm, roughly equivalent to the size of one raisin). Each combination (listed in Table 1) was presented during a daily 20-trial session. Each session started with the opportunity for the subject to smell and look at the two different foods. The experimenter waited for the lemur to sit in front of him before presenting the two items, one in each hand, hands side by side. Then he moved his hands 30 cm apart. When the lemur pointed at one hand through the mesh, it received the food held in that hand as a reward. The reward’s relative positions (left or right) were randomly determined for each trial with the constraint that each food was presented an equal number of times in each position inside a session and the same position was never used more than three consecutive times.

**Table 1:** Percentage of choice of the different food items presented in combination.

	Fig vs.				Raisin vs.			Apple vs.		Carrot vs.
Subjects	Raisin	Apple	Carrot	Pellet	Apple	Carrot	Pellet	Carrot	Pellet	Pellet
Champagne										
N	20	20	20	20	40	40	20	60	20	20
%	90	100	100	90	82.5	82.5	95	31.7	95	85
Z	3.35***	4.25***	4.25***	3.35***	4.27***	4.27***	3.8***	−2.71**	3.8***	2.91**
Crumble										
N	20	20	20	20	20	80	40	40	20	20
%	100	85	95	90	90	71.3	82.5	82.5	95	80
Z	4.25***	2.91**	3.8***	3.35***	3.35***	3.69***	3.95***	3.95***	3.8***	2.46*
Clément										
N	20	20	20	20	100	20	20	60	20	20
%	95	95	95	90	61	90	100	70	80	95
Z	3.8***	3.8***	3.8***	3.35***	2.1*	3.35***	4.25***	2.97**	2.46*	3.8***
Hiro										
N	20	20	20	20	40	20	60	60	20	20
%	80	80	75	80	75	75	65	66.7	85	75
Z	2.46*	2.46*	2.01*	2.46*	3.00**	2.01*	2.19*	2.45**	2.91**	2.01*

* p<0.05,

** p<0.01,

*** p<0.001, two-tailed binomial test.

After individual preferences being established, the lemurs were tested on a reverse-reward contingency task using combinations of two food items, a more and a less preferred item. At the beginning of a trial, the apparatus was placed 40 cm away from the mesh. The experimenter placed his closed hands (containing the items) at the centre of the tray, then moved them apart simultaneously to deposit the rewards on the center of the two white circles at each extremity of the tray. The tray was maintained away from the cage during 3 s to allow the subject to look at the items before making its choice. The experimenter checked that the two sides were visually explored by the subject before moving the tray forward, 25 cm away from the mesh. After the subject selected a food array, the experimenter pivoted the tray 45° in order to make the non-selected food accessible. A choice was defined as the extension of an arm through the mesh in the direction of one of the items. The inter-trial interval was approximately 10 s, the time necessary for the subject to eat the reward. A daily session consisted of 10 trials only, in order to keep the lemurs motivated and focused on the task. If a subject did not complete a session, the trials left were postponed until the next session. The criterion for successful performance was set at 80% of correct responses over a block of five sessions (50 trials).

#### Statistical analysis

Significance was established using a two-tailed binomial test, with the probability of choosing either food being .5. The significance level was set at .05.

### Results

#### Food preferences

Food preference tests revealed the same preference order for three of the four subjects (Clément, Hiro and Crumble). This order was fig > raisin > apple > carrot > pellet and agreed with preferences in other brown lemurs [12]. The remaining subject, Champagne, showed a slightly different preference order: fig > raisin > carrot > apple > pellet; her preference for carrot over apple was robust across sessions (Table 1). We presented subjects with their second and fourth preference to avoid any bias that might be too strong or too weak and hamper lemurs’ performance. Thus, we used carrot and raisin for Clément, Hiro and Crumble, apple and raisin for Champagne.

#### Reverse-reward task

Due to individual differences in willingness to participate, the number of trials varied between subjects. Champagne realized 1050 trials, Crumble 950, Clément 600 and Hiro 650.

**Figure 1:**
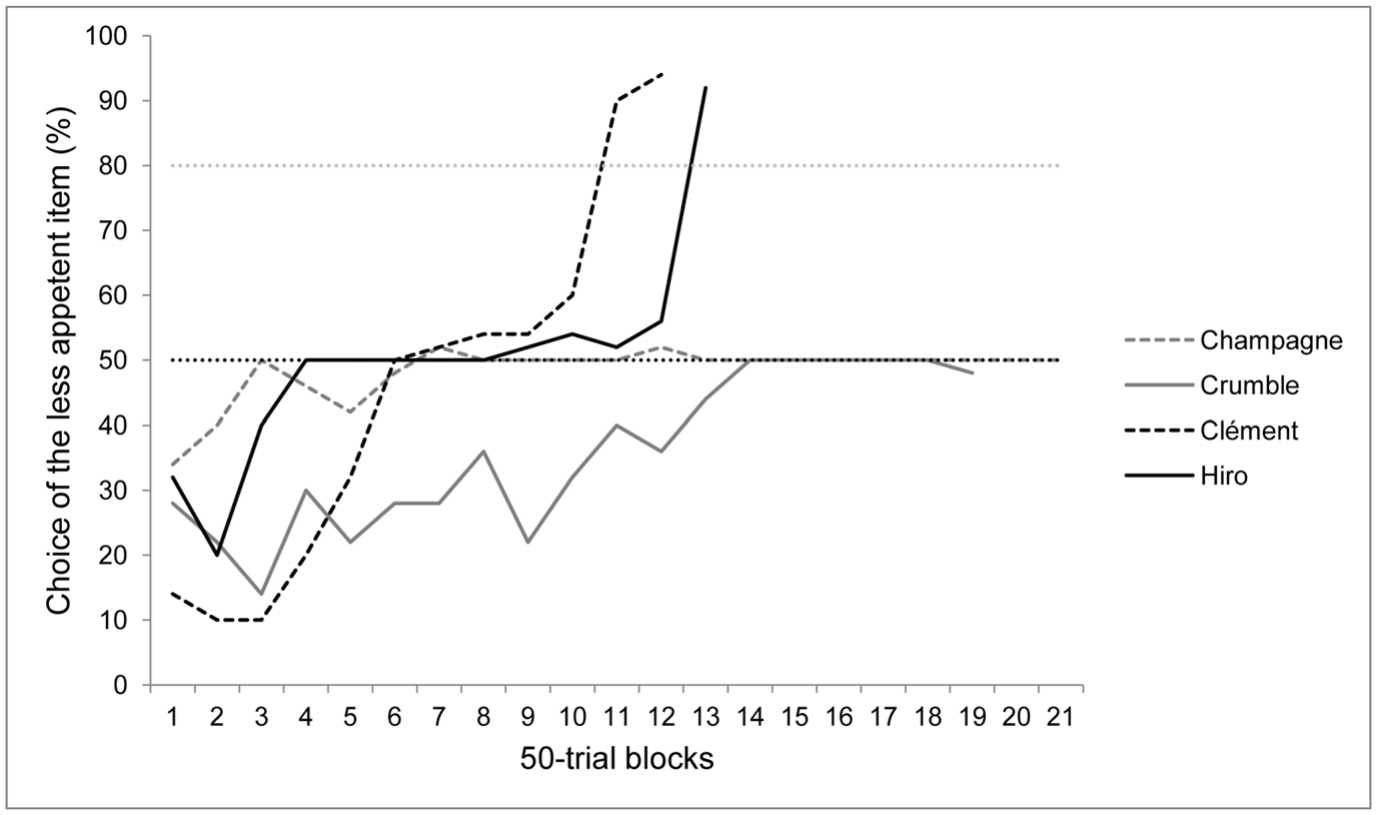
Percentage of choice of the less appetent food across blocks of 50 trials. Grey dotted line: criterion (80%), black dotted line: chance level (50% trials less appetent food selected).

Over the first 50 trials, all four lemurs selected the most attractive food, and thus received the less preferred one as a reward (Champagne: 66% of the trials, Z = −2.12, p<0.05; Crumble: 72%, Z = −2.97, p<0.01; Clément: 86%, Z = −4.95, p<0.001; Hiro: 68%, Z = −2.40, p<0.05). Only one subject (Champagne) showed a slight side preference for the right side (68%, Z = 2.40, p<0.05). Over the last 50 trials, Clément and Hiro significantly selected the less preferred food, respectively choosing it on 94% (Z = 6.08, p<0.001) and 92% (Z = 5.8, p<0.001) of the trials and thus reaching criterion. The remaining two subjects showed no preference (choice of the most attractive item: Champagne: 50%, Z = 0, NS; Crumble: 48%, Z = −0.14, NS). These two subjects showed a clear bias toward the right item, selecting it respectively on 100% (Z = 6.93, p<0.001) and 98% of the trials (Z = 6.65, p<0,001). The complete picture of lemurs’ performances is shown in Figure 1.

There was no correlation between the relative preference for the preferred item vs. non-preferred one (% of choice of one item vs. another one) and the learning rate (number of sessions necessary to reach chance level) of each subject (Spearman correlation test: N = 4, r_s_ = 0.40, NS).

Although only two subjects out of four succeeded, our results demonstrate that lemurs can master the qualitative version of the reverse- reward contingency. It is noteworthy that in a previous study testing lemurs on the quantitative version of the task, only one out of five subjects succeeded after 1300 trials [13].

## Experiment 2

In Experiment 1, two subjects learned to overcome their prepotent choice for the most preferred reward. This result could be explained by simply learning to systematically avoid selecting raisin or choose carrot in order to obtain raisin instead of more generally choosing the less attractive food to obtain the most preferred one. The second experiment assessed the lemurs’ ability to generalize their previous learning to novel combinations.

### Method

#### Procedure

The two successful lemurs (Clément and Hiro) served as subject. The procedure differed from Experiment 1 in that the presented pairs were different combinations of the reward: fig vs. raisin, fig vs. apple, raisin vs. apple, raisin vs. pellet, apple vs. carrot and carrot vs. pellet. Only one combination was presented in a session and each pair was presented over five 10- trial sessions.

### Results and Discussion

**Figure 2:**
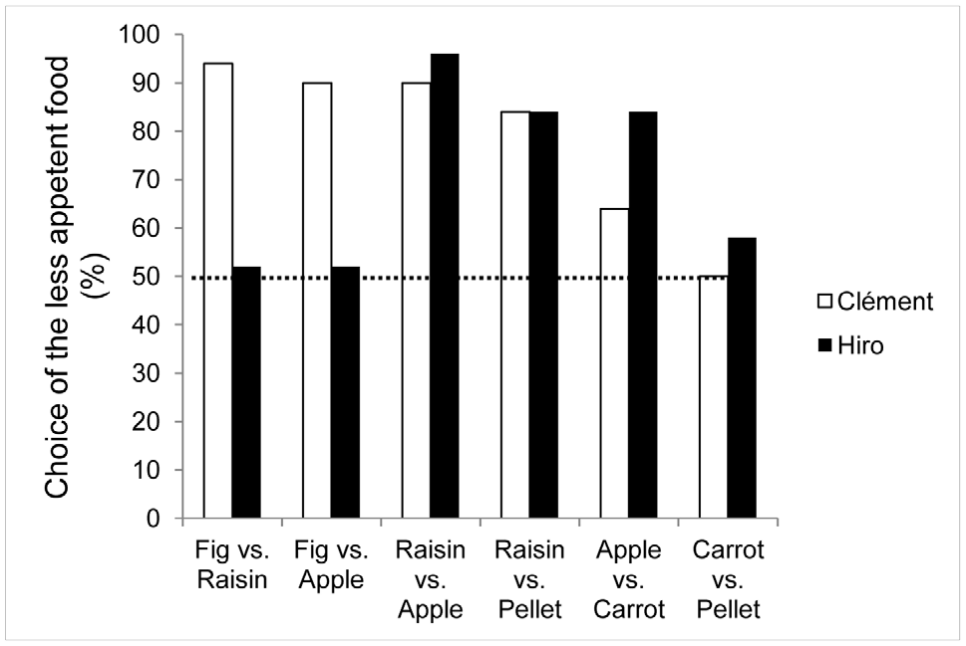
Percentage of choice of the less appetent item (50 trials per combination). Dotted line: chance level (50% trials less appetent food selected).

Over all 50 trials (Fig. 2), Clément selected the less preferred food on 79% of the trials (Z = 9.87, p<0.001) and the right side on 52% (Z = 0.64, NS). Similarly, Hiro selected the less attractive food on 71% of the trials (Z = 7.22, p<0.001), but also showed a significant preference for the left side (73%, Z = −7.91, p<0.001).

Clément selected the less preferred food significantly more often with the following pairs: fig vs. raisin (94%, Z = 6.08, p<0.001), fig vs. apple (90%, Z = 5.52, p<0.001), raisin vs. apple (90%, Z = 5.52, p<0.001) and raisin vs. pellet (84%, Z = 4.67, p<0.001). On these trials, he showed no significant side bias, choosing the right side respectively on 48% (Z = −0.14, NS), 44% (Z = −0.71, NS), 60% (Z = 1.27, NS) and 50% (Z = 0, NS) of the trials. He demonstrated no food preference on the apple vs. carrot (64%, Z = 1.84, NS) and carrot vs. pellet trials (50%, Z = 0; NS). Only on the apple vs. carrot performance he showed a side preference (right side on 70% of the trials, Z = 2.69, p<0.01), he selected the right side only on 40% of the carrot vs. pellet trials (Z = −1.27, NS).

Hiro presented a significant preference for the less preferred food only with raisin vs. apple (96%, Z = 6.36, p<0.001), raisin vs. pellet (84%, Z = 4.67, p<0.001) and apple vs. pellet trials (84%, Z = 4.67, p<0.001), with no side preference on these trials (respectively choosing the right side on 50%, Z = 0, NS; 34%, Z = −2.12, NS; and 58% of the trials, Z = 0.99, NS). He showed no preference on the fig vs. raisin (52%, Z = 0.14, NS), fig vs. apple (52%, Z = 0.14, NS) or carrot vs. pellet trials (58%, Z = 0.99, NS); these latter choices revealed a strong bias for the left side of the apparatus: he selected the right item on 6% (Z = −6.08, p<0.001), 2% (Z = −6.65, p<0.001) and 12% of the trials (Z = −5.23, p<0.001).

With novel pairings, fewer than 50 trials were needed to start selecting the less preferred food item and it was selected more often from the first session with pairs for which the subjects were successful (Table 2), indicating that the subjects were not still learning the rule but had generalized it. Hereto, there was no correlation between the relative preferences for the different items and the subjects’ performances (% of choices of the less preferred food) (Spearman correlation test; Clement: r_s_ = −0.15, Hiro: r_s_ = −0.68, NS).

**Table 2:** Percentages of choice of the less preferred item on the first and second sessions for the pairs for which the subjects succeeded.

		Fig vs.		Raisin vs.		Apple vs.	Carrot vs.
Subject	Session	Raisin	Apple	Apple	Pellet	Carrot	Pellet
Clément	Session 1	90%	80%	70%	60%	80%	30%
	Session 2	100%	100%	90%	90%	60%	30%
Hiro	Session 1	50%	60%	90%	50%	90%	50%
	Session 2	50%	50%	100%	70%	90%	40%

The lemurs’ ability to generalize the previously learned rule to novel pairs of food indicates they did not simply learn to react to a fixed context with carrot and raisin as choices. The lemurs were able to use a more general, context-free rule and rapidly transferred the knowledge they had acquired during the previous learning phase. Although both subjects transferred the previously learned rule, they did not do so to the same extent. Clément generalized to four out of six novel pairs, compared to three pairs by Hiro. Also noteworthy is the fact that these pairs were not exactly the same for the two lemurs. Clément transferred the rule to the pairs involving fig and raisin as the preferred food items, Hiro to the pairs with raisin and apple. This may be due to better self-control abilities by Clément who could have shown more resistance to incentive properties of fig, the most attractive food presented during the pretests.

Two explanations may account for individual differences recorded between the two subjects’ ability to generalize to novel pairs. First, these two subjects differ in age, Clément being the oldest male in the group and Hiro being the youngest. Age is known to affect impulsivity in human and non human primates [30], [31]. The differences recorded with low-attractive rewards may reflect a difference in motivation for obtaining the most preferred food. Thus, even if the order of preference did not differ between the two lemurs, their absolute attractiveness might have done, which could also explain the difference in performance when highly prized items were used. Nevertheless, there was no correlation between the relative preferences for the different items and the subject’s performances. One way to evaluate these individual differences would be to test how many pieces of less preferred food would be needed for it to be chosen over a single piece of the most attractive food (see [12]).

## Experiment 3

Given that lemurs could generalize the rule to novel pairs of food, we tested their ability to master the task in the classical reversed reward contingency task, using the same food item (raisin) in various quantities**.** We wanted to see if contrary to capuchin monkeys, lemurs would transfer the rule from quality to quantity which represents another dimension of the task.

### Methods

#### Subjects, housing and apparatus

The subjects were the two lemurs (Clément and Hiro) who had mastered the task in the first experiment. Their housing conditions and the apparatus used during the test sessions were exactly the same as in Experiment 1.

#### Procedure

The subjects were presented with different quantities of raisin with decreasing ratio (in the following order): 1 vs. 4, 2 vs. 6, 2 vs. 4 and 3 vs. 5. Evidence from previous studies indicated that lemurs had an initial prepotent preference for the greater reward [11]. Only one combination was presented in each session and five 10-trial sessions were conducted with each combination.

### Results and Discussion

**Figure 3:**
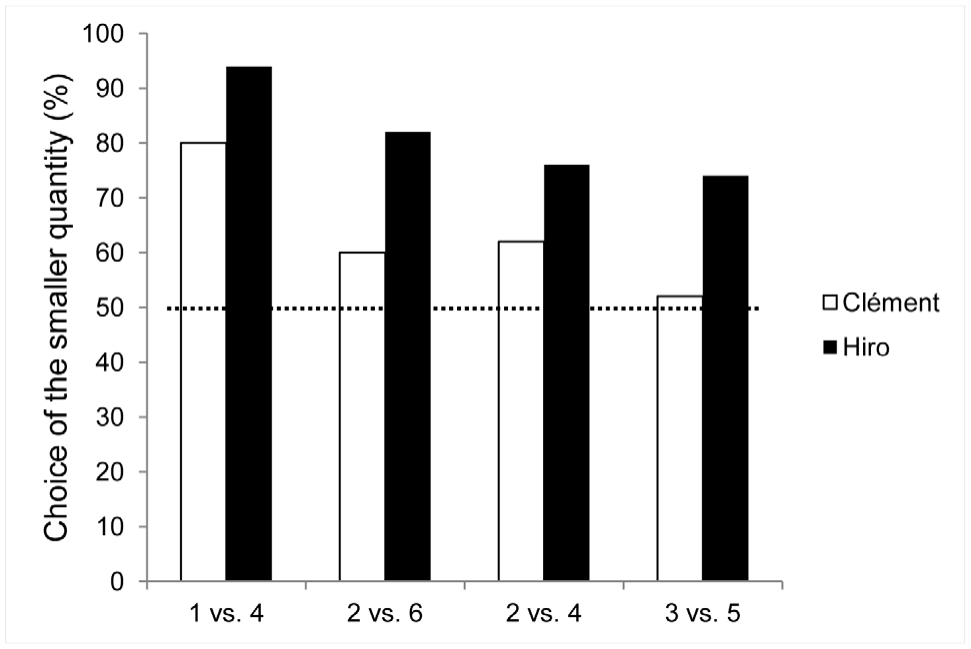
Percentage of choice of the smaller quantity (50 trials per pair of two different quantities). Dotted line: chance level (50% trials smaller quantity selected).

The lemurs performed well above chance (Fig. 3): Clément selected the smaller array on 64% of trials (Z = 3.75, p<0.001) and Hiro on 82% of trials (Z = 8.84, p<0.001).

Taking each pair of quantities separately, Clément chose the smaller arrays only at the highest ratio: 1 vs. 4 trials (80% choice of the 1, Z = 4.1, p<0.001). He showed no significant preference when presented with the three other pairs, selecting the smaller quantity on 60% of trials of 2 vs. 6 (Z = 1.27, NS), 62% of 2 vs. 4 (Z = 1.55, NS) and 52% of 3 vs. 5 trials (Z = 0.14, NS). Hiro selected the smaller array significantly more often in each condition: 94% for 1 vs. 4 (Z = 6.08, p<0.001), 82% for 2 vs. 6 (Z = 4.38, p<0.001), 76% for 2 vs. 4 (Z = 3.53, p<0.001) and 74% for 3 vs. 5 trials (Z = 3.25, p<0.01).

Both subjects’ performances were correlated negatively with the food ratio (defined as the disparity between the two arrays over the average of the two arrays [26]) (Pearson’s correlation test: Clément: r = 0.61; Hiro: r = 0.52, p<0.05).

This experiment demonstrated that one lemur (Hiro) competent on a reversed-reward task with different food qualities could transfer this ability to different quantities of the same food. The second subject only showed some evidence of it. Generalization to the quantitative task seems to depend on the disparity ratio between the two alternatives since the highest ratios elicited the best scores in both subjects.

## Discussion

Although a large variability between individuals was observed, this study confirms that lemurs can master the reversed reward contingency task with different food qualities. Moreover, our results give support to the suggestion by Anderson et al. [27] that the qualitative version of the task could be easier than the conventional one using different food quantities. In the present study, two out of four lemurs mastered the task, whereas only one out of five succeeded in the quantitative version with extensive training [13]. Subjects also learned faster than the lemurs that mastered the quantitative task (requiring on average 600 trials vs. 1500). Furthermore, two individuals generalized the reverse-reward rule to novel pairs of food, indicating that they did not simply learn to select or avoid a specific item but used a more general rule to solve the problem. They also transferred this ability to the conventional task with different quantities of the same food, which is an even more flexible and general use of this rule, flexibility being held to be a *sine qua non* condition for cognitive skills by Tomasello and Call [32]. The reverse transfer (i.e. from quantity to quality) had already been observed in lemurs in a previous study [12], which confirms these primate’s aptitude for switching between modalities for the same type of problem solving.

As pointed out by Anderson *et al*. [27], one possible explanation for the different degrees of difficulty between the two versions of the task may lie in differences in central processing of quantities or qualities. These modalities affect different neurons of the orbitofrontal cortex, a brain area specialized in value assignment [33]. Furthermore, the differences between conditions may involve other brain areas, such as the lateral prefrontal cortex or the *fundus* of the *intraparietal sulcus*, activated during numerical operations. Schifferman [34] argued that reversed reward performance might be explained by three interacting processes: numerical assessment, value assignment and behavioral inhibition **(**which involves the dorso-lateral prefrontal cortex [35], [36]). The qualitative task does not need numerical assessments, which could explain while this version is easier.

Our results, although supporting Anderson *et al*. [27] findings with capuchin monkeys, differ from theirs in some respects. One major difference is that capuchins mastering the task with different food qualities did not show positive transfer to the quantitative task. This discrepancy may be due to moderate attractiveness of the food (sweet potato) used to assess their performance in the quantitative phase, whereas the lemurs were tested with raisin, a highly prized food. Thus motivational differences in the long term might have weakened a possible transfer effect. Nevertheless, this explanation is not supported by the lemurs’ preference for the smaller quantity during the first session of the second phase of the experiment. An alternative explanation is that the rule had been more robustly learned by lemurs because generalization was tested before switching to the quantitative task; thus transfer was more gradual than the abrupt switch from quality to quantity for the capuchins.

An interaction between disparity ratio and performance has been demonstrated in apes for quantities [10], [22], [24], [26]. This disparity ratio effect seems to be advantageous when the task is already mastered (i.e. with numerical arrays for chimpanzees [10], with quantitative trials in the present study, the results for Bobby [26], and experienced apes [22]) and detrimental when it is not (i.e. with candies [10], [22], [26]). Such effect was also suggested in the present study and future researches should directly test this hypothesis by using different pairs of quantities or different types of food across groups to reveal possible changes in speed of mastery of the task.
